# Surface guided radiotherapy practice in paediatric oncology: a survey on behalf of the SIOPE Radiation Oncology Working Group

**DOI:** 10.1093/bjr/tqae049

**Published:** 2024-03-05

**Authors:** Enrica Seravalli, Petra S Kroon, Stephanie Bolle, Cathy Dunlea, Semi B Harrabi, Anne Laprie, Yasmin Lassen-Ramshad, Gillian Whitfield, Geert O Janssens

**Affiliations:** Department of Radiation Oncology, University Medical Center Utrecht, 3508 GA, The Netherlands; Department of Radiation Oncology, University Medical Center Utrecht, 3508 GA, The Netherlands; Department of Radiation Oncology, Gustave Roussy Campus, Villejuif 94 800, France; Department of Oncology, University College London Hospitals NHS Foundation Trust, London NW1 2PB, United Kingdom; Department of Radiation Oncology, University Hospital Heidelberg, Heidelberg 69120, Germany; Institut Claudius Regaud, Institut Universitaire du Cancer de Toulouse-Oncopole, Toulouse NeuroImaging Center, Université de Toulouse, Inserm, UPS, Toulouse 31100, France; Danish Centre for Particle Therapy, Aarhus University Hospital, Aarhus DK-8200, Denmark; The Christie NHS Foundation Trust and Division of Cancer Sciences, University of Manchester, Manchester Cancer Research Centre, Manchester Academic Health Science Centre, Manchester M20 4BX, United Kingdom; Department of Radiation Oncology, University Medical Center Utrecht, 3508 GA, The Netherlands; Princess Maxima Center for Pediatric Oncology, Utrecht 3582CS, The Netherlands

**Keywords:** surface guided radiotherapy, paediatrics, image guided radiotherapy

## Abstract

**Introduction:**

Surface guided radiotherapy (SGRT) is increasingly being implemented to track patient’s surface movement and position during radiation therapy. However, limited information is available on the SGRT use in paediatrics. The aim of this double survey was to map SIOPE (European Society for Paediatric Oncology)-affiliated centres using SGRT and to gain information on potential indications, observed, or expected benefits.

**Methods:**

A double online survey was distributed to 246 SIOPE-affiliated radiotherapy (RT) centres. Multiple choices, yes/no, and open answers were included. The first survey (41 questions) was active from February to March 2021. A shortened version (13 questions) was repeated in March 2023 to detect trends in SGRT use within the same community.

**Results:**

Respectively, 76/142 (54%) and 28/142 (20%) responding centres used and planned to use SGRT clinically, including 4/34 (12%) new centres since 2021. Among the SGRT users, 33/76 (43%) already applied this technology to paediatric treatments. The main benefits of improved patient comfort, better monitoring of intrafraction motion, and more accurate initial patient set-up expected by future users did not differ from current SGRT-users (*P* = .893). Among non-SGRT users, the main hurdles to implement SGRT were costs and time for installation. In paediatrics, SGRT is applied to all anatomical sites.

**Conclusion:**

This work provides information on the practice of SGRT in paediatrics across SIOPE-affiliated RT centres which can serve as a basis for departments when considering the purchase of SGRT systems.

**Advances in knowledge:**

Since little information is available in the literature on the use of SGRT in paediatrics, the results of this double survey can serve as a basis for departments treating children when considering the purchase of an SGRT system.

## Introduction

Surface guided radiotherapy (SGRT) uses optical imaging technology to track the patient’s surface movement and position during radiation therapy without additional radiation dose.^1^^,^^2^ A reference surface is used to calculate the correction of the actual patient position in translations and rotations. When the patient’s surface deviates from the reference position above a user-defined tolerance, the treatment beam can be interrupted. SGRT offers imaging with sub-millimetre detectability, real-time performance, availability at all couch angles, and the largest field of view among all clinical imaging modalities.^2^^,^^3^

In recent years, the clinical use of SGRT has increased, demonstrating utility for initial patient positioning^4^^,^^5^ and real-time patient motion monitoring in a variety of anatomical sites. Examples of SGRT applications are breathing motion monitoring in breast deep inspiration breath hold treatment^3^^,^^6^^,^^7^ and locally advanced lung cancer,^8^ or monitoring the patient’s head during non-coplanar treatments.^9^ For targets located in the extremities, SGRT can lead to an improved treatment posture thanks to the extended field-of-view, thereby reducing the need for repositioning, or re-entering the room to adjust the patient, and so reducing the overall time per session.^10^^,^^11^ Moreover, it has been shown that SGRT can improve the efficiency of the radiotherapy (RT) workflow, by reducing the time required to set-up the patient,^12^^,^^13^ and patient safety.^14^

Since 2018, a number of review papers and guidelines have been published covering various aspects of the clinical applications of SGRT, in particular for adult patients.^1^^,^^2^^,^^15–18^ Two surveys reporting on the practice, mainly with adult patients, of this technology in the United States^16^ and Europe^19^ have been published. Information on the SGRT use in paediatrics (patients up to 18 years old) is limited, although SGRT could improve monitoring of the possible intrafraction motion of young patients during treatment. In a case report, the palliative radiation treatment of an 18-month old boy with a relapsed Wilms tumour using SGRT was described.^20^ The patient had a large anterior mediastinal mass which critically obstructed his airway. SGRT treatment could be delivered in a sufficiently short time slot without the need of anaesthesia. Taylor et al investigated the potential role of SGRT for the management of interfractional gastrointestinal gas volume variation in paediatric abdominal RT.^21^ The key idea is that while SGRT would not replace cone beam CT (CBCT) imaging, it could enable a fully personalized Image Guided Radiotherapy (IGRT) schedule for each patient and reduce CBCT imaging to only required fractions. Also, SGRT systems have been recommended as a safety feature in paediatric treatments to assist in patient set-up and provide additional error detection.^14^^,^^22^

The purpose of this study was to map the SGRT practice in paediatric patients across SIOPE (European Society for Paediatric Oncology)-affiliated centres and so to gain more insight into its implementation. An electronic survey was conducted in 2021 and 2023 to identify actual and future SGRT users, any change over time in potential users, the advantages of this technology in clinical practice, the hurdles to implement it and finally to which anatomical sites SGRT is applied.

## Methods

Two online open voluntary questionnaire were designed using Survey Monkey (SVMK Inc., CA, United States) to assess the use and implementation of SGRT across 246 SIOPE-affiliated paediatric RT departments in 35 countries^23^ (https://SIOP-E.eu/about-SIOP-E/members/).

The first survey (41 questions) was active from February to March 2021 (Supplementary Data S1). A shorter version of the first survey (13 questions) was repeated in March 2023, aiming to detect changes in the use of SGRT over time (Supplementary Data S2). The survey length was intended to be brief: 10 min for participants who had surface imaging and less than 5 min for those who did not. Only one responder per institution was asked to fill in and return the survey.

The link to the survey was sent by e-mail and allowed for web-based data entry. The questionnaire announcement can be found in the Supplementary material. No incentives were offered for the participation to the questionnaire.

The survey was developed by a medical physicist expert and radiation oncologist both with vast experience in paediatrics. Survey questions were organized into two parts: the first part focussed on the participant institutional setting, the availability of SGRT and the time since implementation, the potential advantages in clinical practice for actual and future users and the reasons for not implementing this technology among non-users. The second part addressed the clinical use of SGRT, including applications (eg, initial positioning, intrafraction monitoring) and types of treatment (eg, anatomical site). The survey included multiple-choice questions with room for remarks, as well as yes/no and open-ended questions. Depending on the answers given, certain questions were skipped if not applicable. A maximum of two questions per page were included; the first survey had 31 pages, the second had 13. The selection of one response option was enforced. The respondents were able to review and change their answers through a back button. The usability and technical functionality of the electronic questionnaire had been tested before fielding the questionnaire. A unique site visitor was based on the IP address. No cookies were used to assign a unique user identifier to each client computer. The identification of potential duplicate entries was not based on the IP address of the client computer but on the name of the person, and department, filling the questionnaire.

The responses were reviewed to improve data quality, that is, to avoid duplicate, inconsistent, or contradictory answers within the same institution. Questionnaires which terminated early (where, eg, users did not go through all questionnaire pages) were also analysed. Multiple answers from the same institution were concatenated. Answers from non-existent names/cities/facilities were excluded. The answers of the responders that completed both surveys were counted once. If the answers differed between the two surveys, the answers of the most recent survey were considered. Open-ended questions were individually evaluated; similar answers/comments were grouped together.

Data were processed in Excel. The view rate and participation rate were computed. The former is the ratio of unique survey and unique site visitors, while the latter the ratio of unique visitors who agreed to participate and unique first survey page visitors.^24^ The differences in expected and observed SGRT benefits were assessed by the two-sided Wilcoxon signed-rank test using paired comparison in SPSS version 25 (IBM corporation) and statistical significance was defined as *P* < .05.

## Results

**Figure 1.**
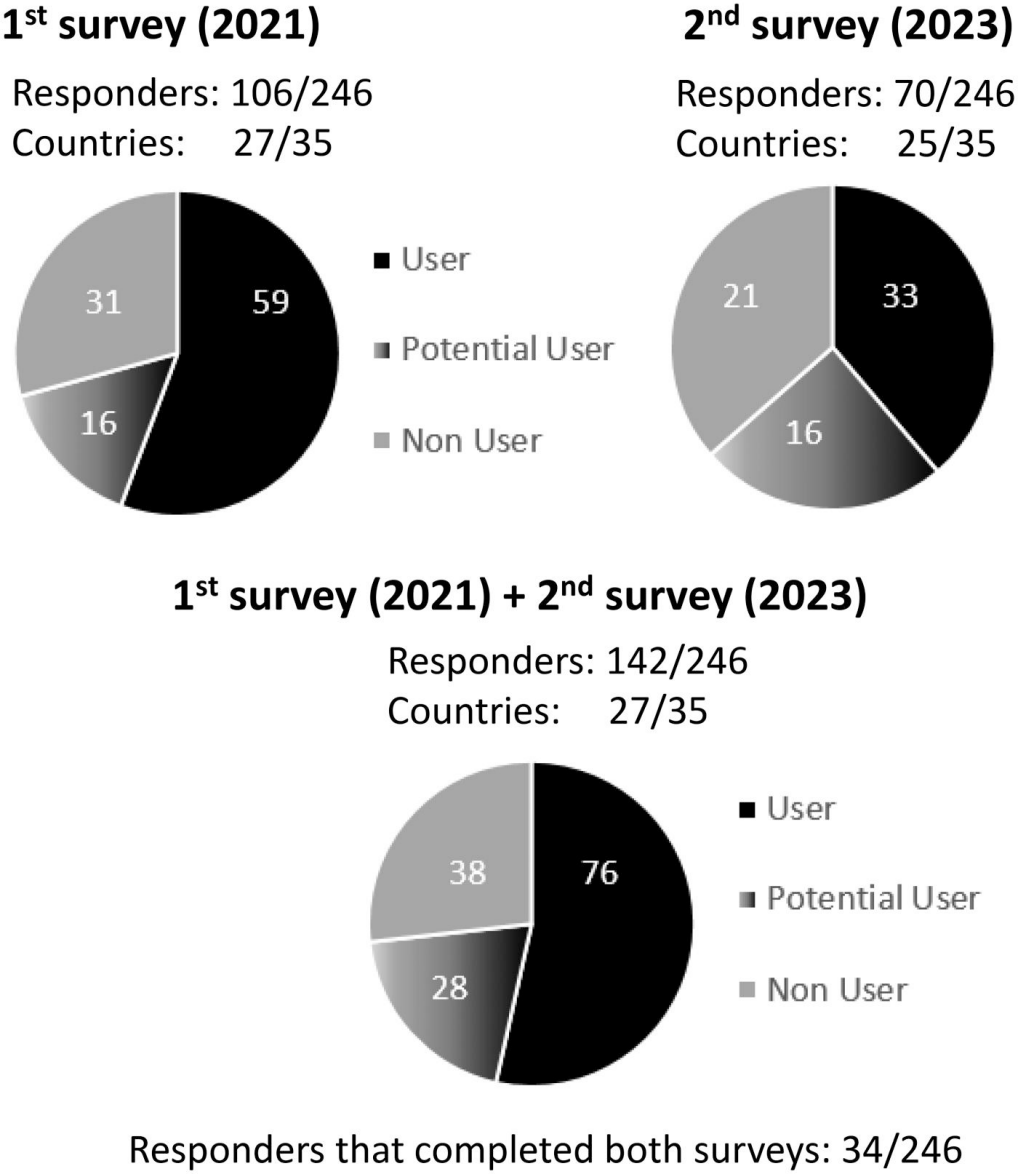
Overview of the survey’s responders. Radiation oncologists working in the 246 SIOPE-affiliated paediatric RT departments were invited to participate and returned the survey.

The view rate was 99% and 100% for the 1st and 2nd survey, while the participation rate was 63% and 96%, respectively. An overview of the survey’s responses, completed by radiation oncologists, is given in Figure 1. In the following sections, the results are based on the respondents who completed the first and second survey, unless stated otherwise.

**Figure 2.**
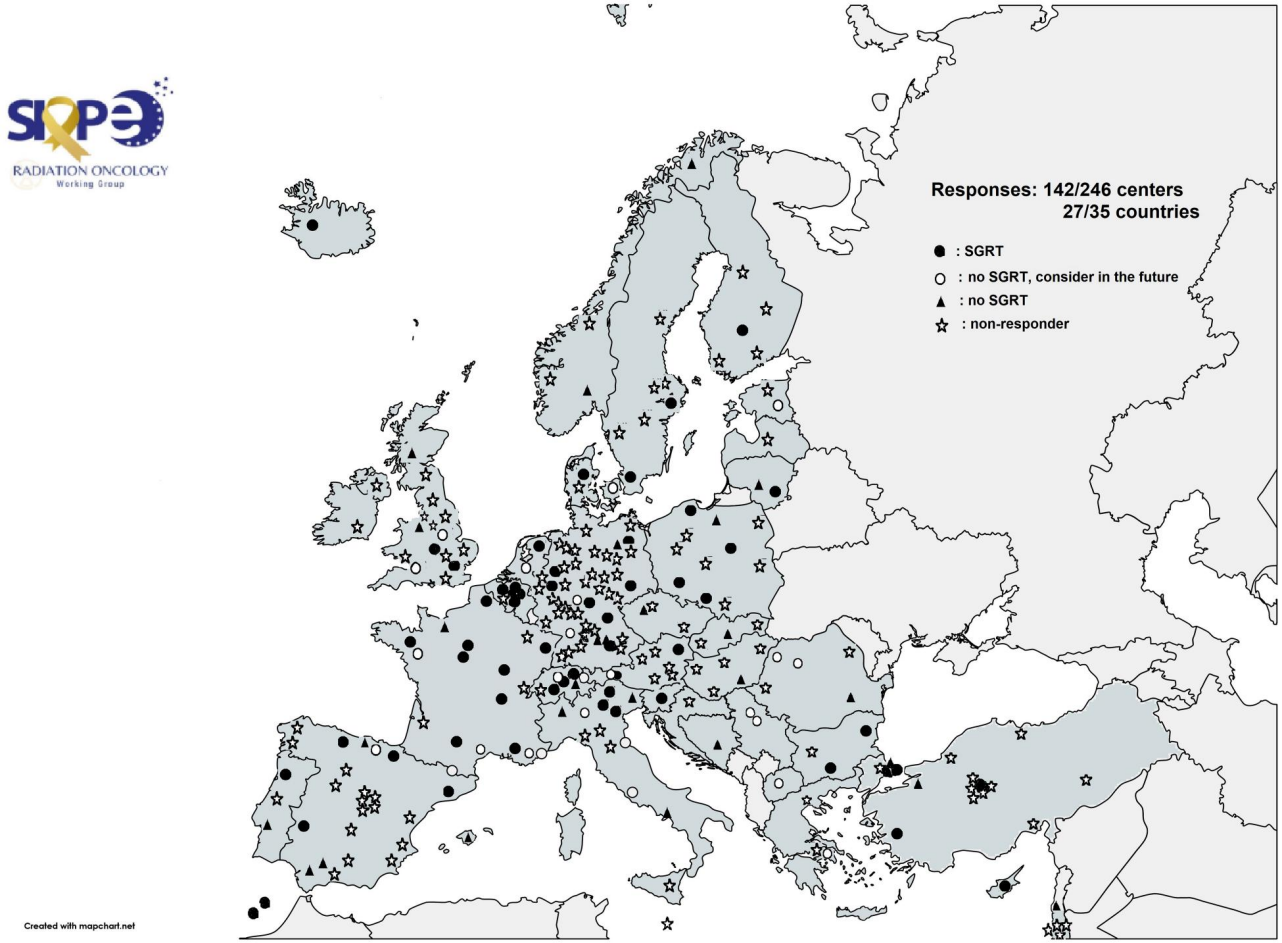
Geographical distribution of the 35 SIOPE-affiliated countries (grey-blue colour) with the 246 invited radiotherapy departments and the different responses (user, no user considering purchase, no user) regarding the status of SGRT in 2021/2023. The map was created with https://www.mapchart.net/.

In Figure 2, the geographical distribution of the responding centres is depicted: 76/142 (54%) of the responders used SGRT clinically, 28/142 (20%) were considering purchasing the technology in the near future, while 38/142 (27%) did not have SGRT and were not considering investing in this technology. Based on the information provided by the 34 responders who completed both questionnaires, 4/34 (12%) additional RT departments implemented SGRT in the clinic between 2021 and 2023.

Among the SGRT users, only 33/76 (43%) apply this technology to paediatric treatments. However, 55% of the other centres are planning to expand the use of SGRT to children soon. The majority (55%) of the SGRT users not applying this technology to paediatrics (43/76) is planning to expand its use for this patient category, while 27% and 18% of these departments do not apply it to paediatrics because of no (expected) benefit of SGRT above the IGRT technology already used for paediatrics and the limited patient numbers.

**Figure 3.**
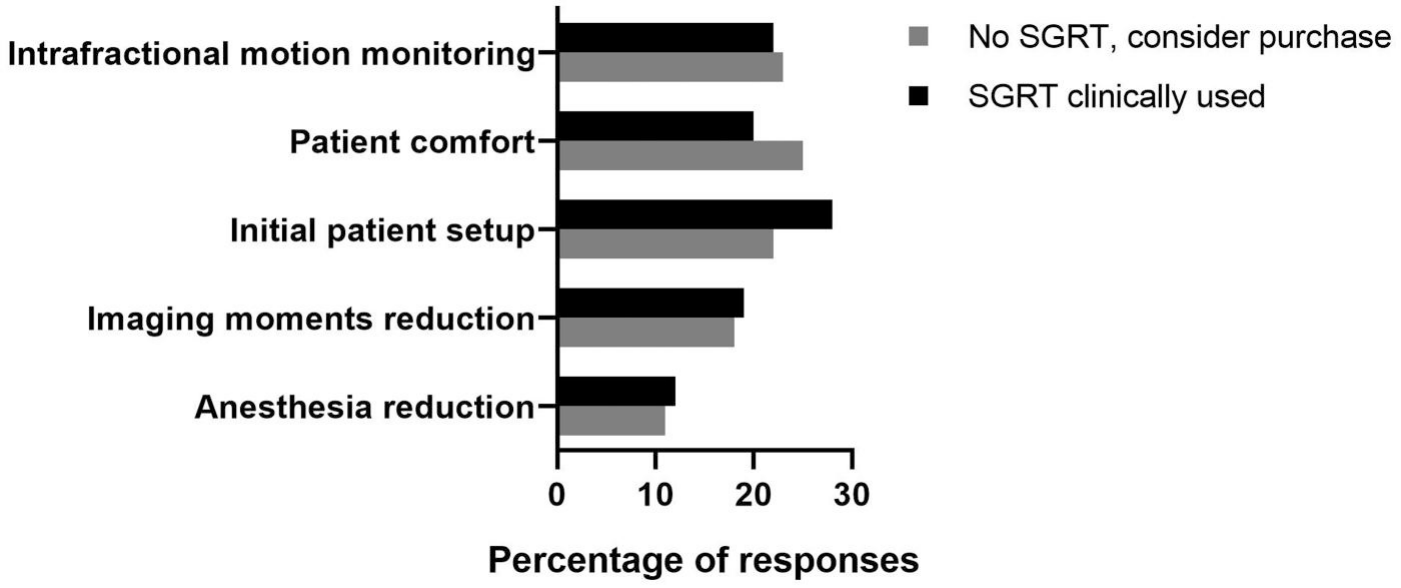
Expected and observed benefits of SGRT in paediatrics among the SIOPE responders who are considering the acquisition of an SGRT system (28/142) and the SGRT users (76/142), respectively.

Expected benefits in paediatrics among the responders who are considering the purchase of surface imaging technology (28/142) are improved patient comfort (25%), better monitoring of the intrafraction motion (23%) and more accurate initial patient set-up (22%) (Figure 3). Similar results were found when asking current SGRT users (76/142) for the observed benefits in children of this technology in daily practice (*P* = .893).

In paediatrics, SGRT is applied to all anatomical sites with the highest frequency for target volumes located in the thorax (25%) followed by the abdomen (24%), pelvis (17%), extremities (14%), CNS (14%), and head and neck (6%).

**Figure 4.**
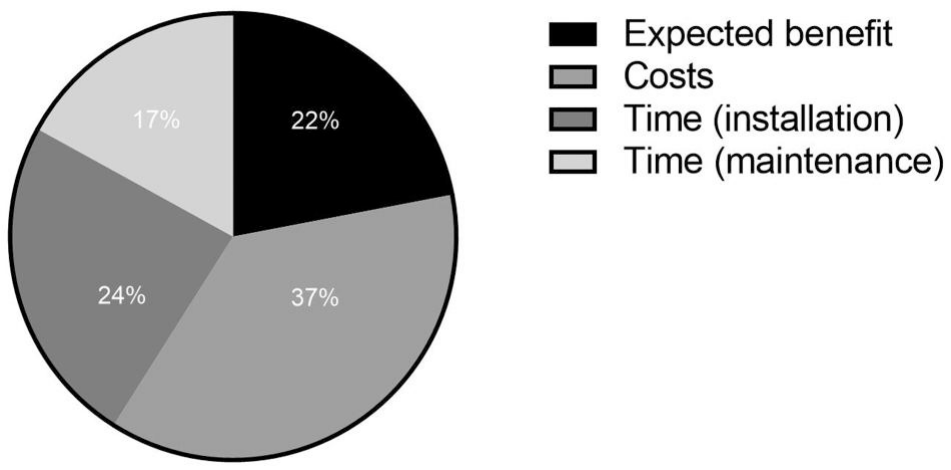
Hurdles to implement SGRT among the 38 SIOPE centres not intending to invest in SGRT.

Among the non-SGRT users, 22% do not expect a benefit of this technology above the IGRT technology already available in the clinic. The main hurdles to implement this technology were costs (37%) and time for installation (24%), as shown in Figure 4.

For 94/104 (90%) of the users and the potential users, SGRT can significantly improve the daily workflow of paediatric RT.

Relevant remarks in the free text box, made by the SGRT users, concerned the inability to use blankets and towels during treatment potentially resulting in patient discomfort and temperature drop for those requiring anaesthesia. In contrast to adults, children can consciously interact with the SGRT system, for example by moving on purpose, interrupting the treatment delivery. This issue should be taken into account when instructing the patient on the treatment. Several responders expressed the need for a guideline dedicated to paediatrics for this technology.

## Discussion

This report, based on a survey performed in 2021 and repeated in 2023, aimed to assess the practice of SGRT in paediatrics across RT departments located in SIOPE-affiliated countries; 76 departments use SGRT while only 33 of the respondent centres apply this technique in paediatrics. Between 2021 and 2023, four additional departments implemented SGRT showing the growing interest in the clinical use of this technology. The main benefits in paediatrics reported by SGRT users are improved patient comfort, better monitoring of intrafraction motion, and more accurate initial patient set-up. Among non-users, the biggest hurdles to implement SGRT in the clinic are time and costs. SGRT is used for all anatomical sites.

The percentage of SIOPE-affiliated centres using SGRT in daily practice is around 54% and is comparable with the percentage reported by similar surveys in adults.^16^^,^^19^ However, the majority of SGRT users are not (yet) applying this technology for paediatric indications although half of current SGRT users are planning to start with children soon. This indicates that the starting group is adult patients probably due to the larger number of cases.^23^ It is expected that a modification of the existing guidelines^1^^,^^18^ including recommendations for paediatrics could accelerate its implementation for this group.

This survey has demonstrated that the benefits expected by future SGRT-users are in line with the experiences of current users. Current SGRT-users observe in paediatrics improved patient comfort due to open face mask treatments and the omission of (permanent) skin markers. Tattoo-less RT is really an advantage for paediatrics as the process of getting these tattoos or permanent set-up marks can be traumatizing for a young cohort.^25^ The use of maximal open face masks makes treatment more comfortable while increasing patient compliance without the need for anaesthesia.^26–28^ Other benefits are a more accurate initial patient set-up, as well as better monitoring of intrafraction motion. These benefits correspond to the advantages seen during applications in adult treatments.^11^^,^^19^ The improved initial patient set-up has the potential to decrease the frequency of verification images, which could reduce set-up time and minimize imaging dose exposure (for CBCT, the dose per image ranges from 1 up to 3 mGy depending on the anatomical region that needs to be imaged^29^). For internal treatment sites, the target position is not always well represented by a surface image,^30^ therefore SGRT is often considered a complement to radiographic image guidance. However, a recent study showed that SGRT may also be able to detect gastrointestinal variations triggering adaptive RT pathways.^21^ For deep-seated target volumes, especially those moving independently of bony anatomy, other imaging techniques using CBCT or MRI are generally more accurate for localization.^6^ However, SGRT can reduce target localization uncertainty due to intrafraction motion by the real-time monitoring of the patient surface.

Users are little convinced of a reduction in the use of general anaesthesia using SGRT. This benefit may be difficult to assess outside of a study context since logistics do not allow an anaesthesia team to be available *ad-hoc* just and only in case of poor compliance.

Nonetheless, based on the survey results, it appears that the field of experts involved in irradiating paediatric patients is not convinced about the benefits provided by SGRT. In addition to modify the current guidelines,^1^^,^^18^ there is an urgent need to advocate for greater adoption and use of this technology for childhood radiation therapy.

Some limitations in the survey are recognized. (1) Although the participation rate of both rounds was in line with other similar questionnaires,^16^^,^^19^ fewer centres responded to the second survey compared to the first survey. (2) The geographical distribution of the responders was not uniform across countries, so results may not reflect the situation within all SIOPE-affiliated countries. (3) While the analysis of the results was based on the CHERRIES checklist, the creation of the survey was not.^24^ (4) The answers may be biased by the vision of the professional who returned the survey and therefore it possibly not represents the vision of the whole RT team involved in paediatrics. (5) The nuances of patient comfort, e.g. physical, mental, were not differentiated in the questions of the two surveys. As a consequence from the results it cannot be extracted for which aspect of patient comfort SGRT has the most added value. (6) Considering the fast adoption of this technology in recent years, SGRT practice in paediatrics may change quickly in the near future. Nevertheless, this survey has provided valuable insights into the availability, indications and hurdles in the use of SGRT across the SIOPE-affiliated RT departments at the moment of writing.

## Conclusions

This work provides an overview of the status of SGRT in paediatrics across SIOPE-affiliated centres in 2023. The presented results focusing on paediatric treatments can serve as a basis for departments considering investing in SGRT systems.

## Supplementary Material

tqae049_Supplementary_Data
