# Supplementary material for: Surface guided radiotherapy practice in paediatric oncology: a survey on behalf of the SIOPE Radiation Oncology Working Group
Source: Br J Radiol. 2024 Mar 5;97(1157):1044–9. doi: 10.1093/bjr/tqae049 (PMC11075983; doi:10.1093/bjr/tqae049)
Supplement: tqae049_Supplementary_Data [file tqae049_supplementary_data.zip › tqae049_Supplementary_Data/Supplementary material 1.docx]

**Announcement survey February 2021**

Dear colleague paediatric radiation oncologist,

We would like to invite you to respond to a survey on the use of SGRT in paediatrics across the SIOPE-affiliated countries. The survey is endorsed by the SIOPE radiation oncology working group.

The growing recognition of SGRT as a promising imaging technique has supported its recent spread in a large number of radiation oncology facilities. However, SGRT-based intra-fraction monitoring of paediatric treatments is not widely used and literature is limited. So, the aim of this survey is to map the current SGRT practice in paediatrics that can serve as a basis for departments considering to invest in SGRT systems.

The following questions will take about 10 minutes to complete and will greatly help us to get an overview of the actual status of SGRT for children.

We do realize that a significant number of centers does not use or is not planning to use SGRT. For this reason, we created a mini-survey with just a couple of questions, accessible via the same link.

Kindly note that only one response per centre/hospital is required, ideally by the person in charge of paediatric radiation oncology.

The outcome of this survey will be presented at the virtual SIOPE Annual Meeting (Valencia, April 28-30, 2021) and therefore, we would very much appreciate your response by February 22.

For any other questions that you may have, please contact XXX.


Thank you very much and looking forward to your response to the survey.

Yours sincerely,

XXX

**Announcement survey March 2023**

Dear colleague,

During the annual SIOPe meeting in Valencia, more specific on May 9, a mini-symposium on the use of surface-guided radiotherapy (SGRT) in pediatrics will be organized including a debate on the role of SGRT in relation to IGRT.

In preparation of this symposium, we would like to repeat a short survey with only 2 to 6 questions (5-10 minutes, depending on your response) to get the current status on the use of SGRT in pediatrics across the SIOPe affiliated radiotherapy departments.

Please fill in the survey even if SGRT is not available at your department.

Thanks in advance for answering the survey, and I hope to see a lot of you in Valencia.

Kind regards,

XXX
