# Supplementary material for: Surface guided radiotherapy practice in paediatric oncology: a survey on behalf of the SIOPE Radiation Oncology Working Group
Source: Br J Radiol. 2024 Mar 5;97(1157):1044–9. doi: 10.1093/bjr/tqae049 (PMC11075983; doi:10.1093/bjr/tqae049)
Supplement: tqae049_Supplementary_Data [file tqae049_supplementary_data.zip › tqae049_Supplementary_Data/supplementary material_2_survey 2021.pdf]

Surface guided radiation therapy (SGRT) for paediatrics - current status

Dear colleague paediatric radiation oncologist,

We would like to invite you to respond to a survey on the use of SGRT in paediatrics across the SIOPE-affiliated countries. The survey is endorsed by the SIOPE radiation oncology working group.

The growing recognition of SGRT as a promising imaging technique has supported its recent spread in a large number of radiation oncology facilities. However, SGRT-based intra-fraction monitoring of paediatric treatments is not widely used and literature is limited. So, the aim of this survey is to map the current SGRT practice in paediatrics that can serve as a basis for departments considering to invest in SGRT systems.

The following questions will take about 10 minutes to complete and will greatly help us to get an overview of the actual status of SGRT for children.
We do realize that a significant number of centers does not use or is not planning to use SGRT. For this reason, we created a mini-survey with just a couple of questions, accessible via the same link.

Kindly note that only one response per centre/hospital is required, ideally by the person in charge of paediatric radiation oncology.

The outcome of this survey will be presented at the virtual SIOPE Annual Meeting (Valencia, April 28-30, 2021) and therefore, we would very much appreciate your response by February 22.

For any other questions that you may have, please contact anne.blondeel@siope.eu.

Thank you very much and looking forward to your response to the survey.

Yours sincerely,

SIOP Europe
the European Society for Paediatric Oncology

Surface guided radiation therapy (SGRT) for paediatrics - current status

General questions

* 1. Name of your department or hospital

* 2. City

* 3. Country

SIOP Europe
the European Society for Paediatric Oncology

Surface guided radiation therapy (SGRT) for paediatrics - current status

General questions

* 4. Do you have surface-guided RT **installed or under installation/comissioning** at your department?

- ◯ Yes
- ◯ No

SIOP Europe
the European Society for Paediatric Oncology

Surface guided radiation therapy (SGRT) for paediatrics - current status

General questions

* 5. Does your department consider the **acquisition** of a SGRT system in the next 2 years?

- ◯ Yes
- ◯ No

Surface guided radiation therapy (SGRT) for paediatrics - current status

General questions

6. Please rank the following reasons in order of which they present **hurdles** to implement SGRT in your department (1=most important)

- Costs
- No/limited staff available for installation/commissioning/workflow adaptation
- No clear clinical benefits of SGRT above the IGRT technology already available at your clinic
- Other reason(s)

7. Other factors/reasons? please specify

Surface guided radiation therapy (SGRT) for paediatrics - current status

General questions

8. Do you routinely use image guidance (**IGRT**) (daily imaging, adaptive workflow...) during paediatric treatments?

- ◯ Yes
- ◯ No

Surface guided radiation therapy (SGRT) for paediatrics - current status

9. When already making use of IGRT during treatment, do you expect a **benefit** of SGRT for paediatric patients?

- ◯ Yes
- ◯ No
- ◯ Never thought about it

Any other comments

[ ]

SIOP Europe
the European Society for Paediatric Oncology

Surface guided radiation therapy (SGRT) for paediatrics - current status

General questions

* 10. What **additional benefit** do you expect of SGRT for paediatric patients when already using IGRT? (multiple answers possible)

- [ ] Improving patient comfort (open face mask treatments, no skin markers, ...)
- [ ] Intra-fraction monitoring
- [ ] More accurate initial patient setup
- [ ] Reducing the use of anesthesia
- [ ] Reducing the number of imaging moments for the verification of the patient position over the whole treatment course

Other (please specify)

SIOP Europe
the European Society for Paediatric Oncology

Surface guided radiation therapy (SGRT) for paediatrics - current status

Daily practice

11. Are you already **using** the system **clinically** for paediatrics?

- ◯ Yes
- ◯ No, under installation/commissioning

Surface guided radiation therapy (SGRT) for paediatrics - current status

Daily practice

12. Since **how many years** do you use SGRT in routine practice for paediatric patients?

- ◯ 1 year
- ◯ 2 years
- ◯ 3 years
- ◯ 4 years
- ◯ 5 years
- ◯ 5+ years

Surface guided radiation therapy (SGRT) for paediatrics - current status

Daily practice

* 13. Do you intend to use SGRT for **initial positioning** of paediatric patients?

- [ ] Yes
- [ ] No

Surface guided radiation therapy (SGRT) for paediatrics - current status

Daily practice

* 14. For which **anatomical site(s)** are you planning to use SGRT to position the paediatric patient? (multiple answers possible)

- [ ] Brain
- [ ] Head and Neck
- [ ] Thorax
- [ ] Abdomen
- [ ] Pelvis
- [ ] Extremities
- [ ] Other (please specify)

Surface guided radiation therapy (SGRT) for paediatrics - current status

Daily practice

15. Do you intend to use SGRT for **respiration monitoring** (i.e. breath-hold monitoring, gated delivery,...) of paediatric patients?

- ◯ Yes
- ◯ No

SIOP Europe
the European Society for Paediatric Oncology

Surface guided radiation therapy (SGRT) for paediatrics - current status

Daily practice

* 16. For which of the following **tumor locations/types** are you planning to use respiration monitoring (i.e. breath hold, gating) (multiple answers possible)

- [ ] PTV upper mediastinum
- [ ] PTV lower mediastinum
- [ ] PTV lung metastasis (whole lung RT excluded)
- [ ] PTV lung metastasis (whole lung RT included)
- [ ] Other (please specify)
- [ ] PTV spleen or liver
- [ ] PTV flank
- [ ] PTV whole abdomen

Surface guided radiation therapy (SGRT) for paediatrics - current status

Daily practice

17. Do you plan to use SGRT for **intrafraction monitoring** of paediatric patients?

- ◯ Yes
- ◯ No

SIOP Europe
the European Society for Paediatric Oncology

Surface guided radiation therapy (SGRT) for paediatrics - current status

Daily practice

* 18. For which **anatomical site(s)** are you planning to use SGRT to monitor intra-fraction motion? (multiple answers possible)

- [ ] Brain
- [ ] Head and Neck
- [ ] Thorax
- [ ] Abdomen
- [ ] Pelvis
- [ ] Extremities
- [ ] Other (please specify)

Surface guided radiation therapy (SGRT) for paediatrics - current status

Daily practice

* 19. Do you use SGRT for **initial positioning** of paediatric patients?

- [ ] Yes
- [ ] No

SIOP Europe
the European Society for Paediatric Oncology

Surface guided radiation therapy (SGRT) for paediatrics - current status

Daily practice

* 20. For which **anatomical site(s)** do you use SGRT to position the patient? (multiple answers possible)

- [ ] Brain
- [ ] Head and Neck
- [ ] Thorax
- [ ] Abdomen
- [ ] Pelvis
- [ ] Extremities
- [ ] Other (please specify)

Surface guided radiation therapy (SGRT) for paediatrics - current status

Daily practice

21. Do you use SGRT for **respiration monitoring** (i.e. breath-hold monitoring, gated delivery,...) of paediatric patients?

- ◯ Yes
- ◯ No

Surface guided radiation therapy (SGRT) for paediatrics - current status

Daily practice

* 22. For which of the following **tumor locations/types** do you use respiration monitoring (i.e. breath hold, gating) (multiple answers possible)

- [ ] PTV upper mediastinum
- [ ] PTV lower medistinum
- [ ] PTV lungs metastasis (whole lung RT excluded)
- [ ] PTV lungs metastasis (whole lung RT included)
- [ ] Other (please specify)
- [ ] PTV spleen or liver
- [ ] PTV flank
- [ ] PTV whole abdomen

SIOP Europe
the European Society for Paediatric Oncology

Surface guided radiation therapy (SGRT) for paediatrics - current status

Daily practice

23. Do you use SGRT for **intrafraction monitoring** of paediatric patients?

- ○ Yes
- ○ No

Surface guided radiation therapy (SGRT) for paediatrics - current status

Daily practice

* 24. For which **anatomical site(s)** do you use SGRT to minitor intra-fraction motion? (multiple answers possible)

- [ ] Brain
- [ ] Head and Neck
- [ ] Thorax
- [ ] Other (please specify)
- [ ] Abdomen
- [ ] Pelvis
- [ ] Extremities

SIOP Europe
the European Society for Paediatric Oncology

Surface guided radiation therapy (SGRT) for paediatrics - current status

Daily practice

25. Has the use of **skin markers** in paediatric patients been replaced by the implementation of SGRT ?

- ◯ Yes
- ◯ No
- ◯ Other (please specify)

Surface guided radiation therapy (SGRT) for paediatrics - current status

Daily practice

26. Was the **use of anesthesia** during treatment delivery reduced by the implementation of SGRT?

- ◯ Yes
- ◯ No

Other (please specify)

[ ]

Surface guided radiation therapy (SGRT) for paediatrics - current status

Daily practice

27. Did SGRT allow the implementation of paediatric treatments with **open face** immobilization mask or mask-less at your department?

- ○ Yes
- ○ No

Other (please specify)

[ ]

Surface guided radiation therapy (SGRT) for paediatrics - current status

Equipment

28. How do you plan to install the SGRT system?

- ◯ All linacs of the deparment
- ◯ Selected number of linacs

Other

[ ]

* 29. Which SGRT **vendor** will be installed in your department?

- ☐ Brain-Lab
- ☐ C-RAD
- ☐ Varian Identify/Humediq
- ☐ Vision RT/OSMS
- ☐ Other Vendor

Surface guided radiation therapy (SGRT) for paediatrics - current status

Equipment

30. Is an SGRT system installed on?

- ○ All linacs of the deparment
- ○ Selected number of linacs

Other

[ ]

* 31. Which SGRT **vendor** do you have installed in your clinic?

- ☐ Brain-Lab
- ☐ C-RAD
- ☐ Varian Identify/Humediq
- ☐ Vision RT/OSMS
- ☐ Other Vendor

SIOP Europe
the European Society for Paediatric Oncology

Surface guided radiation therapy (SGRT) for paediatrics - current status

Final part

32. Do you believe SGRT can significantly **improve the daily workflow** in paediatric RT?

- ◯ Yes
- ◯ No

Surface guided radiation therapy (SGRT) for paediatrics - current status

Final part

33. What kind of **advantages** do you expect from SGRT in paediatrics?
(please rank, 1=most important)

- Improving patient comfort (i.e. open face mask, no skin markers)
- Reducing the use of anesthesia
- Intra-fraction monitoring
- More accurate initial patient setup
- Reducing the number of imaging moments for the verification of the patient position over the whole treatment course
- Other advantage(s)

34. Please specify any other advantages of SGRT not mentioned in the previous question

35. What kind of **challenges** of SGRT do you expect in paediatrics?
(please rank, 1=most important)

- Elevated costs versus limited number of patients
- Complexity of the workflow
- Variable patient anatomy (i.e. interfraction diameter changes)
- Patient compliance
- Other challenge(s)

36. Please specify any other challenges of SGRT for paediatric RT not mentioned in the previous question

37. Are you interested in taking part in an international **working group** on SGRT for paediatrics?

- ◯ Yes
- ◯ No

Surface guided radiation therapy (SGRT) for paediatrics - current status

Final part

You have reached the final question.
Thank you for participating in this survey.

38. Can your name be listed in future reports/publications, as the reference radiation oncologist involved with SGRT for your centre?

- ◯ Yes
- ◯ No

39. Your name and last name?

____

40. Your email address?

____

41. Do you have any further suggestions or comments?

____
