# Supplementary material for: Surface guided radiotherapy practice in paediatric oncology: a survey on behalf of the SIOPE Radiation Oncology Working Group
Source: Br J Radiol. 2024 Mar 5;97(1157):1044–9. doi: 10.1093/bjr/tqae049 (PMC11075983; doi:10.1093/bjr/tqae049)
Supplement: tqae049_Supplementary_Data [file tqae049_supplementary_data.zip › tqae049_Supplementary_Data/supplementary material_3_survey 2023.pdf]

Surface Guided Radiotherapy (SGRT) in pediatrics

Dear colleague,

During the annual SIOPe meeting in Valencia, more specific on May 9, a mini-symposium on the use of surface-guided radiotherapy (SGRT) in pediatrics will be organized including a debate on the role of SGRT in relation to IGRT.

In preparation of this symposium, I would like to repeat a short survey with only 2 to 6 questions (5-10 minutes, depending on your response) to get the current status on the use of SGRT in pediatrics across the SIOPe affiliated radiotherapy departments. Please fill in the survey even if SGRT is not available at your department.

Thanks in advance for answering the survey, and I hope to see a lot of you in Valencia.

Kind regards,

* 1. Please enter your name, institute, country and e-mail adress

Name:

Institute:

Country:

E-mail address:

Surface Guided Radiotherapy (SGRT) in pediatrics

* 2. At your department, do you use SGRT in daily practice?

- ◯ Yes
- ◯ No

Surface Guided Radiotherapy (SGRT) in pediatrics

Surface Guided Radiotherapy (SGRT) in pediatrics

* 3. Does your department consider the acquisition of an SGRT system in the next 2 years?

- ◯ Yes
- ◯ No

Surface Guided Radiotherapy (SGRT) in pediatrics

* 4. Is there a special reason why your department is not planning to invest in SGRT? (multiple answers possible)

- [ ] SGRT technology is too expensive
- [ ] No clear clinical benefit of SGRT is expected
- [ ] No time available for installation
- [ ] No/limited staff available for maintenance or operation
- [ ] Other reasons (please specify in the box)

Surface Guided Radiotherapy (SGRT) in pediatrics

* 5. Which additional benefit do you expect from SGRT for pediatric patients? (multiple answers possible)

- [ ] A reduction in the use of anesthesia
- [ ] A reduction in the imaging moments for the verification of the patient position over the whole treatment course
- [ ] A more accurate initial patient setup
- [ ] An improved patient comfort (open face masks, no skin markers, ...)
- [ ] A better monitoring of the intra-fraction motion
- [ ] Other benefits (please specify in the box)

Surface Guided Radiotherapy (SGRT) in pediatrics

* 6. Was SGRT installed at your department/institute in the past 2 years ?

- ◯ Yes
- ◯ No

Surface Guided Radiotherapy (SGRT) in pediatrics

* 7. Are you already using SGRT for pediatrics in daily practice?

- ◯ Yes
- ◯ No

Surface Guided Radiotherapy (SGRT) in pediatrics

* 8. Is there a special reason why your department is not using SGRT for pediatric patients? (multiple answers possible)

- ☐ The number of pediatric patients in the department is too low to set up SGRT
- ☐ There is no clear benefit of SGRT above the IGRT technology which is already used within the department for pediatrics
- ☐ In the near future, we are planning to expand our SGRT indications including pediatrics.
- ☐ Other reasons

Surface Guided Radiotherapy (SGRT) in pediatrics

* 9. What additional benefit do you expect of SGRT for pediatric patients? (multiple answers possible)

- ☐ A reduction in the use of anesthesia
- ☐ A reduction in the imaging moments for the verification of the patient position over the whole treatment course
- ☐ A more accurate initial patient setup
- ☐ An improved patient comfort (e.g. open face mask treatments, no skin markers, ...)
- ☐ An improved monitoring of Intra-fraction motion
- ☐ Other

* 10. Which benefit do you observe with SGRT in pediatric patients? (multiple answers possible)

- ☐ A reduction in the use of anesthesia
- ☐ A reduction in the imaging moments for the verification of the patient position over the whole treatment course
- ☐ A more accurate initial patient setup
- ☐ An improved patient comfort (e.g. open face mask treatments, no skin markers, ...)
- ☐ An improved monitoring of Intra-fraction motion
- ☐ Other

Surface Guided Radiotherapy (SGRT) in pediatrics

* 11. For which of the following tumor sites do you use SGRT?

- [ ] CNS
- [ ] Head and Neck
- [ ] Thorax
- [ ] Abdomen
- [ ] Pelvis
- [ ] Extremities

Final part

* 12. Can your name be listed in future reports/publications, as the reference radiation oncologist involved in SGRT for your center?

- ◯ Yes
- ◯ No

Surface Guided Radiotherapy (SGRT) in pediatrics

13. You have reached the final question. Thank you for participating in this survey.

Do you have further comments or suggestions?
